# Hybrid spatiotemporal modeling of nutrient cycling in wetland ecosystems using advanced mapping techniques and machine learning approaches

**DOI:** 10.1038/s41598-026-40585-5

**Published:** 2026-02-19

**Authors:** Eric Ariel L. Salas, Kellsie Schrack, Sakthi S. Kumaran, Robert Bennett

**Affiliations:** Agricultural Research Development Program (ARDP), Central State University, Wilberforce, OH 45384 USA

**Keywords:** Ecology, Ecology, Environmental sciences, Hydrology

## Abstract

Accurate spatiotemporal monitoring of nutrient cycling in wetlands is critical for conservation. However, traditional field-based methods are often inadequate for capturing the overall dynamics of wetlands. To address this challenge, we developed and validated a two-stage hybrid Random Forest regression framework that seamlessly integrated in-situ water quality data with wetland features and satellite imagery from Sentinel-1 and Sentinel-2 within the Google Earth Engine platform. The framework first models baseline nutrient concentrations using discrete wetland characteristics (stage 1) and then models the resulting spatial residual using continuous satellite-derived predictors (stage 2). We applied the model to predict quarterly nitrogen and phosphorus concentrations over four years (2021–2024) in the Beavercreek Wetlands Greenway (BWG), a mixed-use landscape. The two-stage model demonstrated exceptional predictive performance for both nitrogen (final $\text {r}^{2}$ = 0.90, RMSE = 0.129 mg/L) and phosphorus (final $\text {r}^{2}$ = 0.89, RMSE = 0.007 mg/L). The variable importance analysis revealed divergent predictive pathways: nitrogen concentration was driven by both landscape-level factors (e.g., land use classes, area and perimeter of wetlands, rainfall) and in-stream biophysical conditions captured by remote sensing (e.g., vegetation and SAR indices), whereas phosphorus was controlled by source-loading from developed land uses. We produced spatiotemporal maps to visualize these distinct patterns. The maps revealed that the BWG system is subject to seasonal nitrogen stress but has experienced significant recovery from prior phosphorus impairment. This study offers a diagnostic framework that could inform focused wetland management strategies and aid in monitoring the health and function of wetland ecosystems.

## Introduction

Wetlands have been known to purify water, sequester carbon, and provide habitat biodiversity^1,2^. However, more than 35% of the global wetlands have already disappeared since 1970^3^. This loss threatens one of their most important functions: the nitrogen (N) and phosphorus (P) nutrient cycle that maintains the water quality in connected aquatic systems^4^. As agricultural runoff and urban development increase the load of nutrients in surface waters^5^, processing of nutrients from wetland becomes more problematic^6^.

The cycling of nutrients in wetlands depends on the way morphology, water flow, and surrounding land use^6,7^ interact. These interactions result in varying nutrient processing rates in wetland areas that field sampling cannot capture efficiently^8^. Although traditional point measurements provide detailed information, they also require much labor and time. In addition, they are ineffective in showing how nutrients move through entire wetland systems. When measuring how effective wetlands clean water or predicting how they respond to changing conditions, using traditional methods can be problematic^9^.

Researchers have started to combine field measurements with remote sensing (RS) and machine learning (ML) models to better understand nutrient patterns^10^. Early work used simple methods to estimate nutrient levels between sampling points. Recent studies have used multiple environmental factors to better explain where nutrients are found^11,12^. These studies show that the cycling of nutrients in wetlands is influenced by variables operating on different scales, from local water chemistry to land use throughout the landscape^13^.

Many ML algorithms have improved the prediction of nutrients in wetlands by analyzing complex relationships between environmental factors and nutrient levels^14^. For instance, the Random Forest (RF) model works for wetland studies because of its ability to handle large amounts of data. Although it identifies non-linear patterns, RF does not make assumptions about the behavior of the data^15^. However, current studies are still struggling to find an efficient way to combine two different types of data^16^. One type describes the general system conditions^17^(e.g., discrete land use percentages, morphological measurements such as wetland area, perimeter, and shapes). The other type captures small-scale spatial differences and spatial environmental conditions within the wetlands^18^ (e.g., continuous satellite images and derivatives). Most studies have examined the cycling of nutrients in wetlands in different regions^8^ and wetland types^19^. However, we did not find studies that developed a method to properly combine discrete and continuous data for a time-series prediction of nutrients in wetlands.

Two-stage modeling approaches have proven useful in other fields for handling complex spatial data^20,21^. These approaches can handle the practical challenge of working with discrete and continuous datasets that operate on different temporal scales^10^. In this study, we developed a two-stage modeling approach to address the challenge of combining different types of data. The first stage of the model predicted nutrients based on general wetland characteristics. The second stage predicted the spatial errors from the first stage using satellite data. We tested this method on nitrogen (N) and phosphorus (P) data from water samples collected over four years in three Beavercreek Wetlands Greenway (BWG) wetlands in Ohio. Specifically, our objectives were to: (1) build models of current nutrient patterns in wetland systems; (2) make quarterly maps showing nutrient levels in high resolution; and (3) compare how environmental factors affect N versus P patterns differently.

## Results

### Efficiency of the RF model

Our hybrid RF approach showed strong predictive performance for both N and P concentrations throughout the two-stage modeling process (Table 1). For the prediction of N, the $\text {r}^{2}$ values improved from 0.86 in stage 1 to 0.88 in stage 2 and achieved a final combined model $\text {r}^{2}$ of 0.90. Consequently, RMSE decreased from 0.249 mg/L in stage 1 to 0.169 mg/L in stage 2 and reached the lowest value of 0.129 mg/L in the final combined model. MAE followed a similar pattern, decreasing from 0.222 mg/L to 0.155 mg/L to 0.116 mg/L in the final model. The values of MBE remained relatively low throughout, which indicated minimal systematic bias in the predictions.

For the prediction of P, the models also showed consistently high accuracy with $\text {r}^{2}$ values of 0.86, 0.87, and 0.89 for stage 1, stage 2, and the final combined model, respectively. RMSE values were notably low in all stages (0.007 mg/L, 0.005 mg/L, and 0.007 mg/L), reflecting the precision of the models in predicting P concentrations. The MAE values were also low, decreasing from 0.005 mg/L in stage 1 to 0.004 mg/L in stage 2, and 0.002 mg/L in the final model. Similarly to N models, the MBE values remained consistently low at 0.003 mg/L, 0.002 mg/L, and 0.002 mg/L.

**Table 1:** Summary of model performance metrics for the two-stage regression framework. The table shows validation statistics for both N and P predictions at each modeling phase: model for discrete variables (stage 1), model for continuous variables (stage 2), and the final combined model.

**Statistics**	**Nitrogen (N)**			**Phosphorus (P)**		
	**Stage 1**	**Stage 2**	**Final**	**Stage 1**	**Stage 2**	**Final**
$\text {r}^{2}$	0.86	0.88	0.90	0.86	0.87	0.89
RMSE	0.249	0.169	0.129	0.007	0.005	0.007
MAE	0.222	0.155	0.116	0.005	0.004	0.002
MBE	-0.015	0.004	-0.052	0.003	0.002	0.002

### Spatial mapping of nitrogen concentrations

**Fig. 1:**
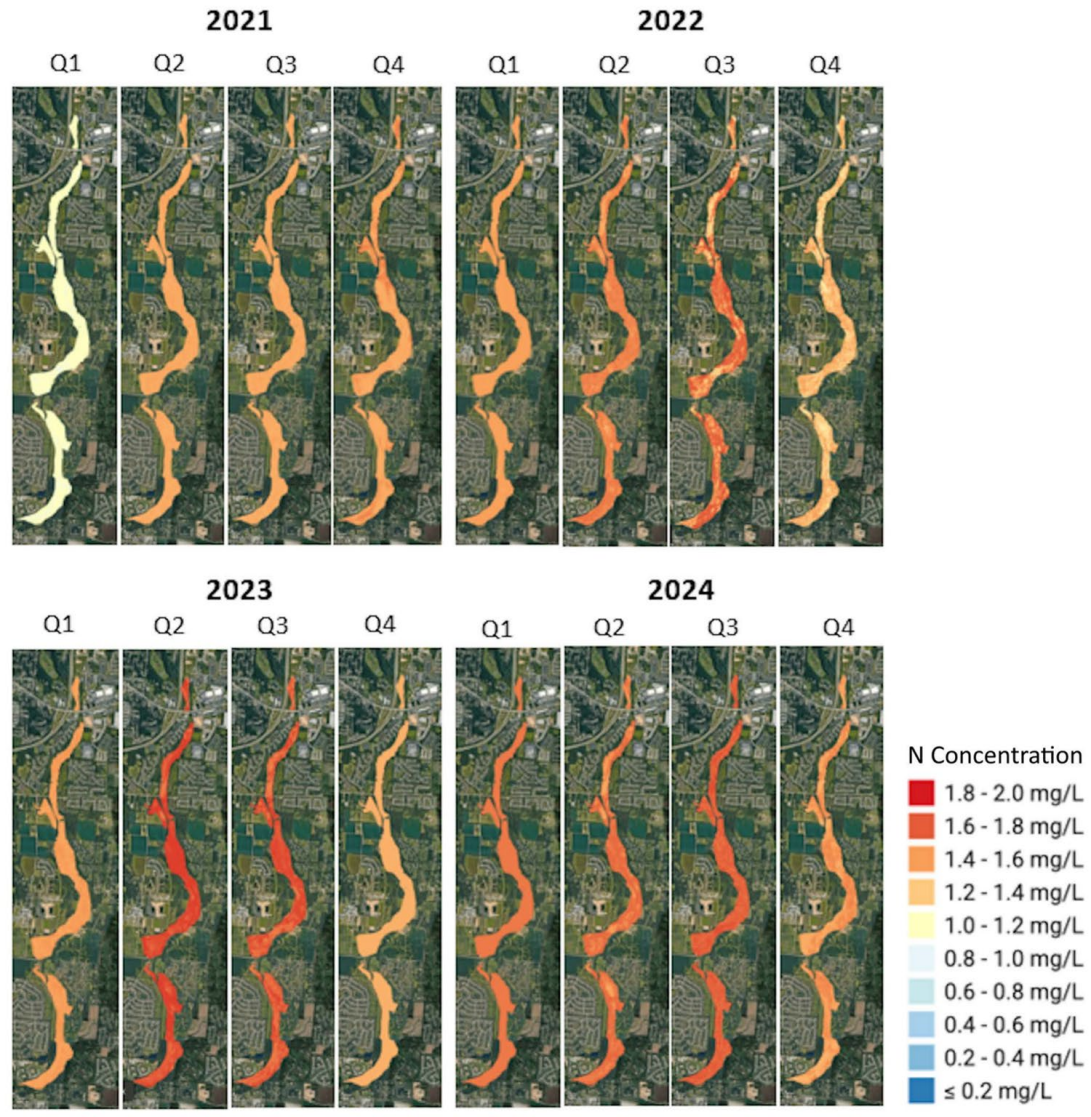
Predicted spatial distribution of N concentrations in the wetlands, presented quarterly from 2021 to 2024. The time-series maps, produced by the two-stage model, revealed significant seasonal and inter-annual variability in N levels. This figure was created in Google Earth Engine (GEE) (https://earthengine.google.com/) and further processed in ArcGIS Pro v.3.4.0 software by Esri (source: https://www.esri.com/). The basemap was provided by ESRI World Imagery (source: ESRI, Maxar, Earthstar Geographics, and the GIS USER Community).

The N maps for the four-year study period (2021-2024) revealed different temporal and spatial patterns within the wetland ecosystem (Fig. 1). In 2021, N concentrations exhibited a seasonal progression, and Q1 showed predominantly low concentrations ($\le$0.2 mg/L to 0.6 mg/L) throughout most of the wetland area. Quarters Q2 and Q3 displayed elevated concentrations ranging from 1.2 mg/L to 1.4 mg/L, and from 1.8 mg/L to 2.0 mg/L in parts of the wetland. Q4 showed a transition back to moderate concentrations in the range of 1.2 mg/L to 1.6 mg/L.

The N map results in 2022 demonstrated a similar seasonal pattern, but we observed higher baseline concentrations compared to 2021. Q1 maintained relatively low concentrations (0.6 mg/L to 1.2 mg/L), while the Q2 and Q3 exhibited the highest levels of N concentration ($\sim$2.0 mg/L) throughout large areas of the wetland. Q4 showed a decrease in moderate to high concentrations (1.4 mg/L to 1.8 mg/L).

The year 2023 showed a notable change in the N pattern, with Q2 and Q3 showing the most dramatic increase in concentration. Q2 and Q3 indicated the highest concentrations of N in the study area (1.8 mg/L to 2.0 mg/L). In contrast, the Q1 and Q4 quarters maintained moderate concentrations (1.2 mg/L to 1.6 mg/L). The results of 2024 showed a more moderate pattern compared to previous years, showing concentrations of 1.0 mg/L to 1.8 mg/L throughout most quarters. We found that the peak concentrations of Q2 and Q3 were less extreme than those in 2022 and 2023, although still elevated compared to the corresponding quarters in 2021.

The comparison of results in the four years showed a gradual increase in N concentrations in Q1 from 2021 to 2024. The 2021 Q1 had the lowest values ($\le$0.2 mg/L to 0.6 mg/L), while 2024 Q1 had moderate concentration values (1.0 mg/L to 1.4 mg/L). We also observed consistently high N concentrations in Q2 and Q3; specifically in 2022 Q2 and 2023 Q2 to Q3 (1.8 mg/L to 2.0 mg/L). Except for slightly lower values in the last quarter of 2021, the concentration levels in Q4 remained stable across years (1.2 mg/L to 1.6 mg/L).

Lastly, we observed consistent seasonal patterns throughout the four years of study, with Q2 and Q3 showing higher N concentrations than Q1 and Q4. Periods 2022 and 2023 showed the most pronounced magnitude of seasonal variation. The difference between quarters with low concentrations (Q1, Q4) and quarters with high concentrations (Q2, Q3) was 1.2 mg/L to 1.4 mg/L (from 0.6 mg/L to 0.8 mg/L in Q1 to 1.8 mg/L to 2.0 mg/L in Q2-Q3). In contrast, 2021 showed the smallest seasonal variation (0.6 mg/L to 0.8 mg/L), with Q1 concentrations around 0.2 mg/L to 0.6 mg/L and Q2-Q3 around 1.2 mg/L to 1.4 mg/L. The year 2024 showed an intermediate seasonal concentration variation (0.8 mg/L to 1.0 mg/L), with Q1 around 1.0 mg/L to 1.2 mg/L and Q2-Q3 around 1.6 mg/L to 1.8 mg/L. The last year showed more gradual transitions between quarters.

### Spatial mapping of phosphorus concentrations

**Fig. 2:**
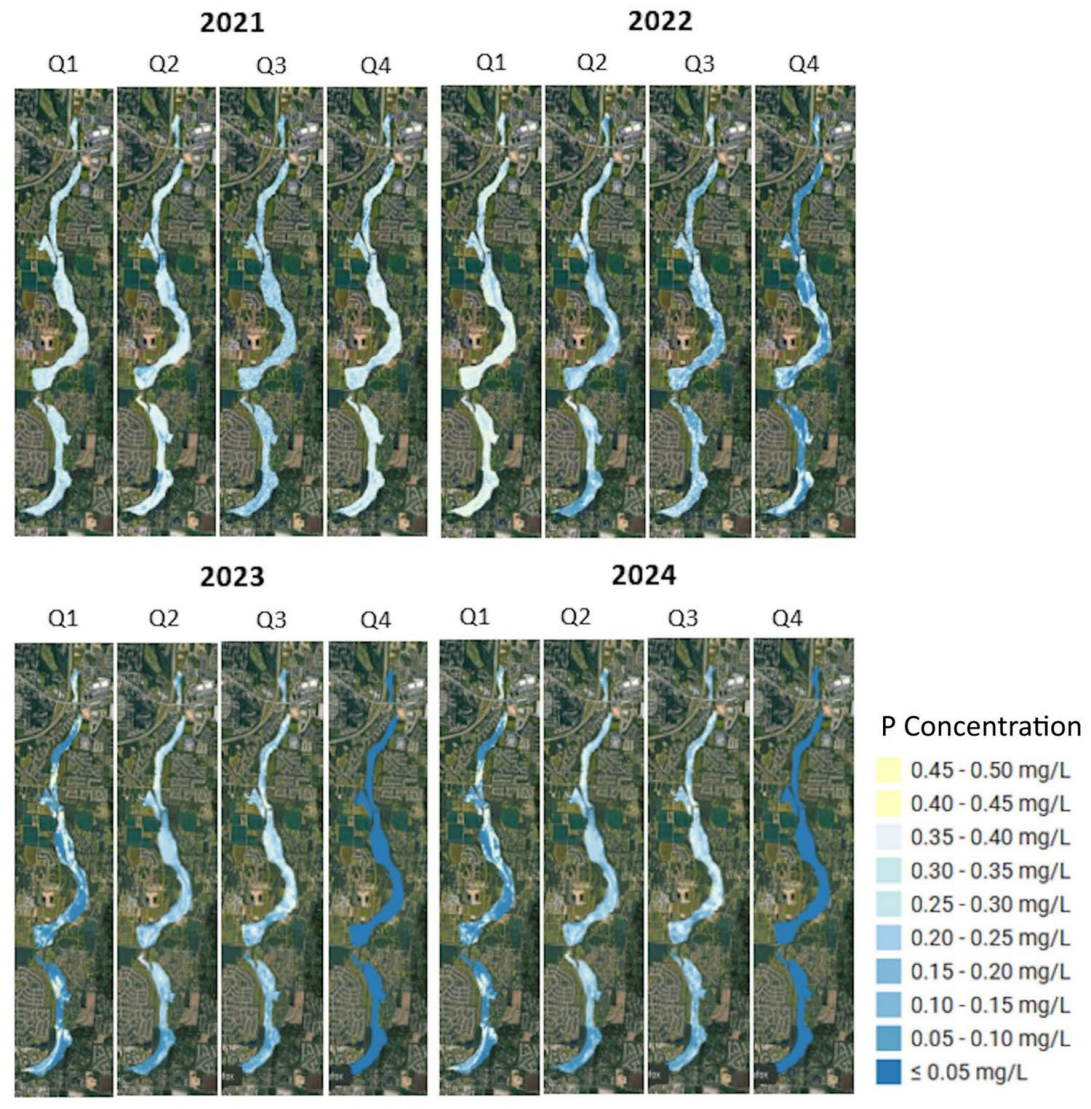
Predicted spatial distribution of P concentrations in the wetlands, presented quarterly from 2021 to 2024. The time-series maps, produced by the two-stage model, revealed significant seasonal and inter-annual variability in P levels. This figure was created in Google Earth Engine (GEE) (https://earthengine.google.com/) and further processed in ArcGIS Pro v.3.4.0 software by Esri (source: https://www.esri.com/). The basemap was provided by ESRI World Imagery (source: ESRI, Maxar, Earthstar Geographics, and the GIS USER Community).

The P concentration maps from 2021 to 2024 (Fig. 2) showed a temporal progression in water quality throughout the study area. The 2021 Q1 map, for example, showed elevated P concentrations throughout the wetland system, ranging from 0.25 mg/L to 0.35 mg/L and higher. This pattern remained stable through Q2 and Q4 of 2021, with minimal variation in P levels.

We observed a significant improvement in water quality in 2022 compared to 2021. We observed a decrease in P concentrations in the 0.15 mg/L to 0.25 mg/L range. This improvement was consistent in all quarters, specifically in Q3 and Q4, with notable reductions in P levels throughout the wetlands.

The resulting P maps for 2023 demonstrated a continued improvement in water quality from 2022. The P concentrations appeared to have further decreased ( 0.10 mg/L to 0.20 mg/L) in most areas. The two quarters, Q1 and Q4, exhibited better seasonal water quality compared to Q2 and Q3. We noticed that the 2023 trend remained favorable compared to 2021 and 2022. Our analysis indicated that the 2024 results had similar trends of P in 2023. Q1 and Q4 showed slightly better water quality than the Q2 and Q3 quarters.

The seasonal pattern of P that we observed in 2024 followed a consistent trend throughout the study period. Winter quarters (Q1 and Q4) generally exhibited lower P concentrations than summer quarters (Q2 and Q3). The spatial distribution showed that while the wetland areas have experienced a general improvement from 2021 to 2024, the greatest reductions in P concentrations were observed during the winter months of 2023 and 2024, particularly in Q1 and Q4 ($\le$0.05 mg/L range).

Lastly, the quarterly maps revealed that the P distribution is not uniform. We observed a persistent P ”hotspot” in the wider middle section of the wetland. For example, during the summer peaks of Q3 in 2023 and 2024, this section consistently displayed the highest concentrations of P, ranging from 0.35 mg/L to more than 0.40 mg/L. This spatial pattern suggested that the middle section could be a key area for internal P loading, which may be due to low-flow conditions of late summer.

### Important variables for mapping nutrients

**Fig. 3:**
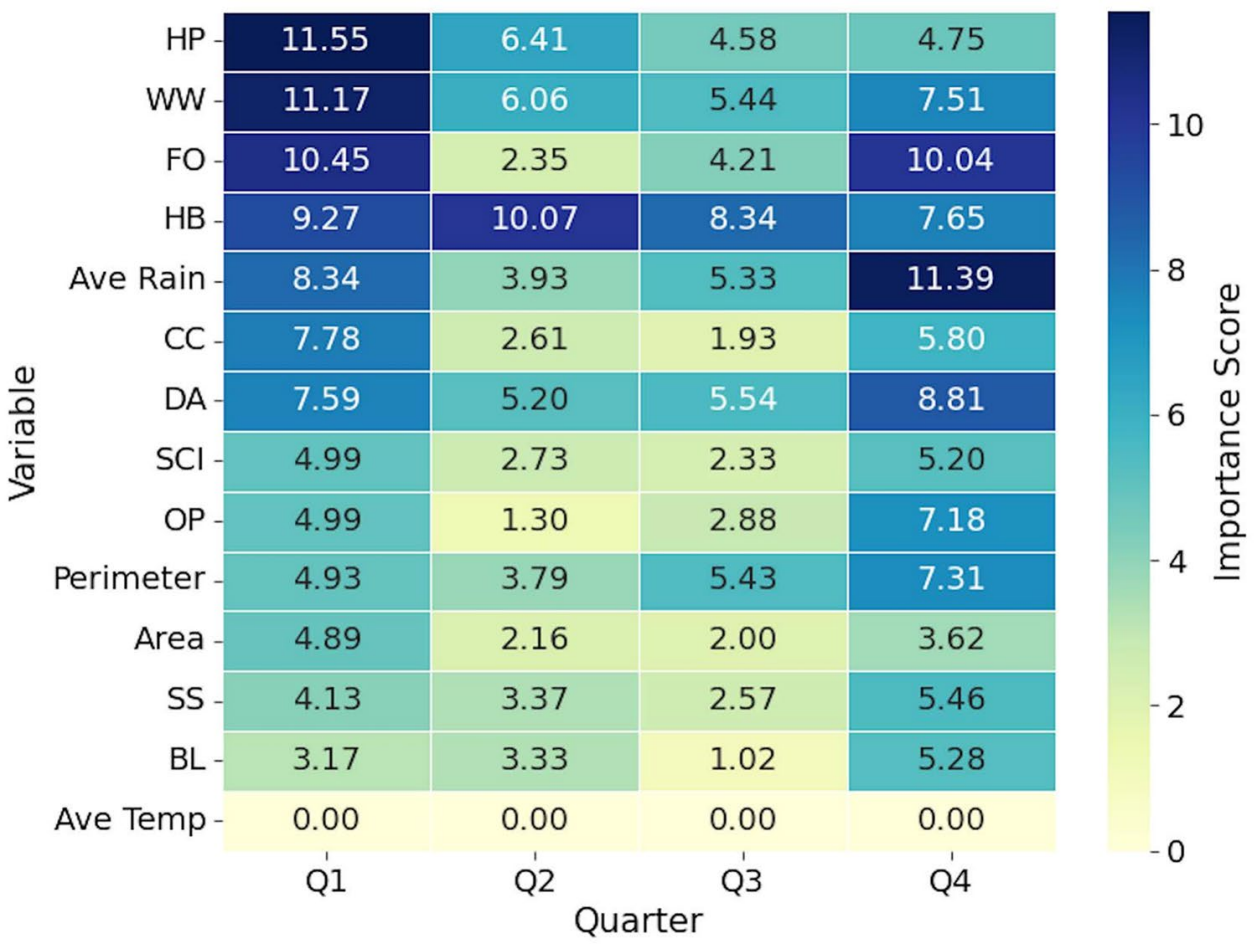
Heatmap showing the 4-year averaged importance scores by quarter in stage 1 for variables used in the estimation of N concentration. *DA* developed area, *CC* cultivated crop, *FO* forest, *WW* woody wetland, *BL* barren land, *OP* open water, *SS* shrub scrub, *HB* herbaceous, *HP* hay/pasture, *SCI* Shape Complexity Index.

**Fig. 4:**
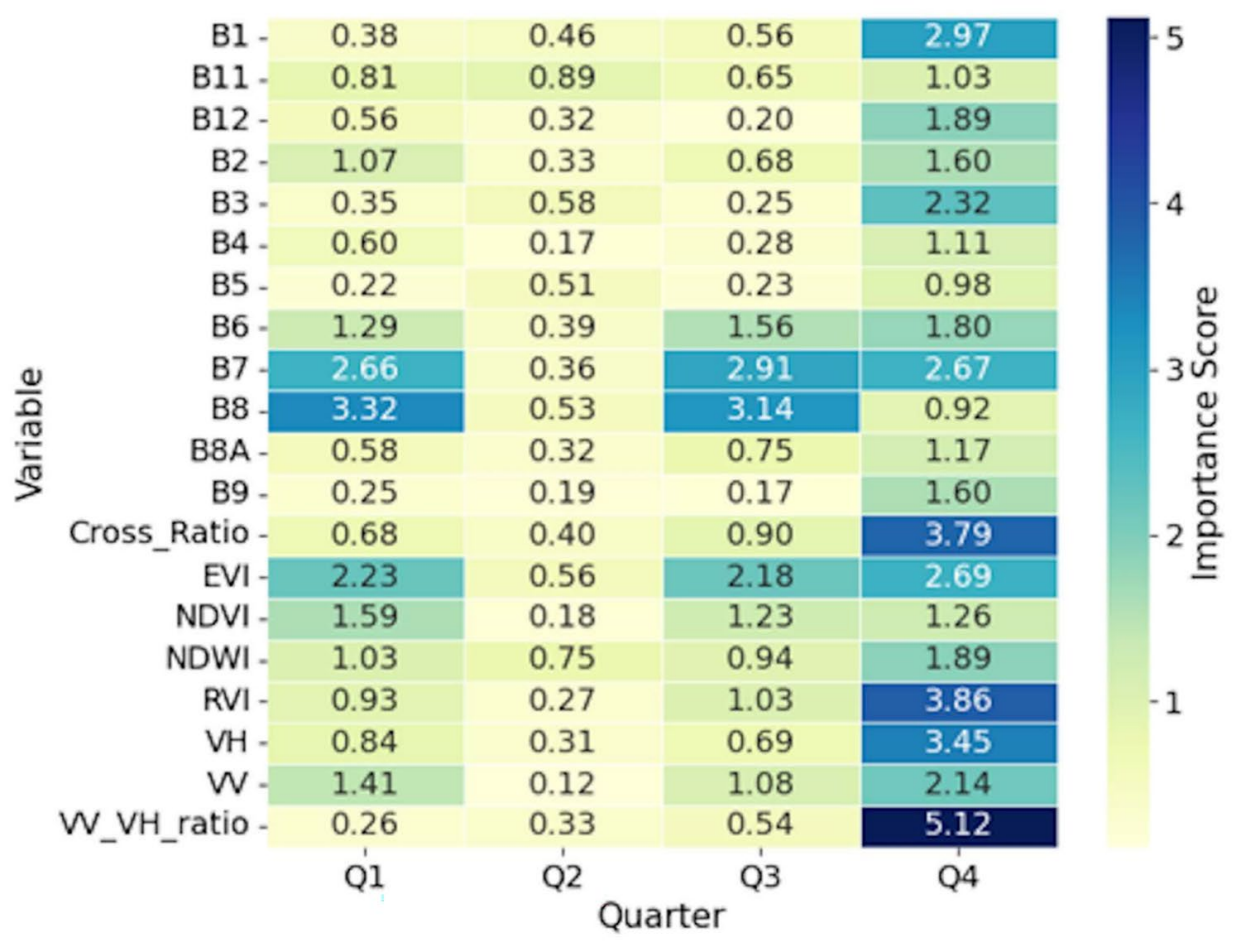
Heatmap showing the 4-year averaged importance scores by quarter in stage 2 for variables used in the estimation of N concentration. *B1 to B12* Sentinel-2 bands, *NDVI* Normalized Difference Vegetation Index, *NDWI* Normalized Difference Water Index, *EVI* Enhanced Vegetation Index, *RVI* Simplified Radar Vegetation Index, *VH* Vertical transmit/Horizontal receive, *VV* Vertical transmit/Vertical receive.

**Fig. 5:**
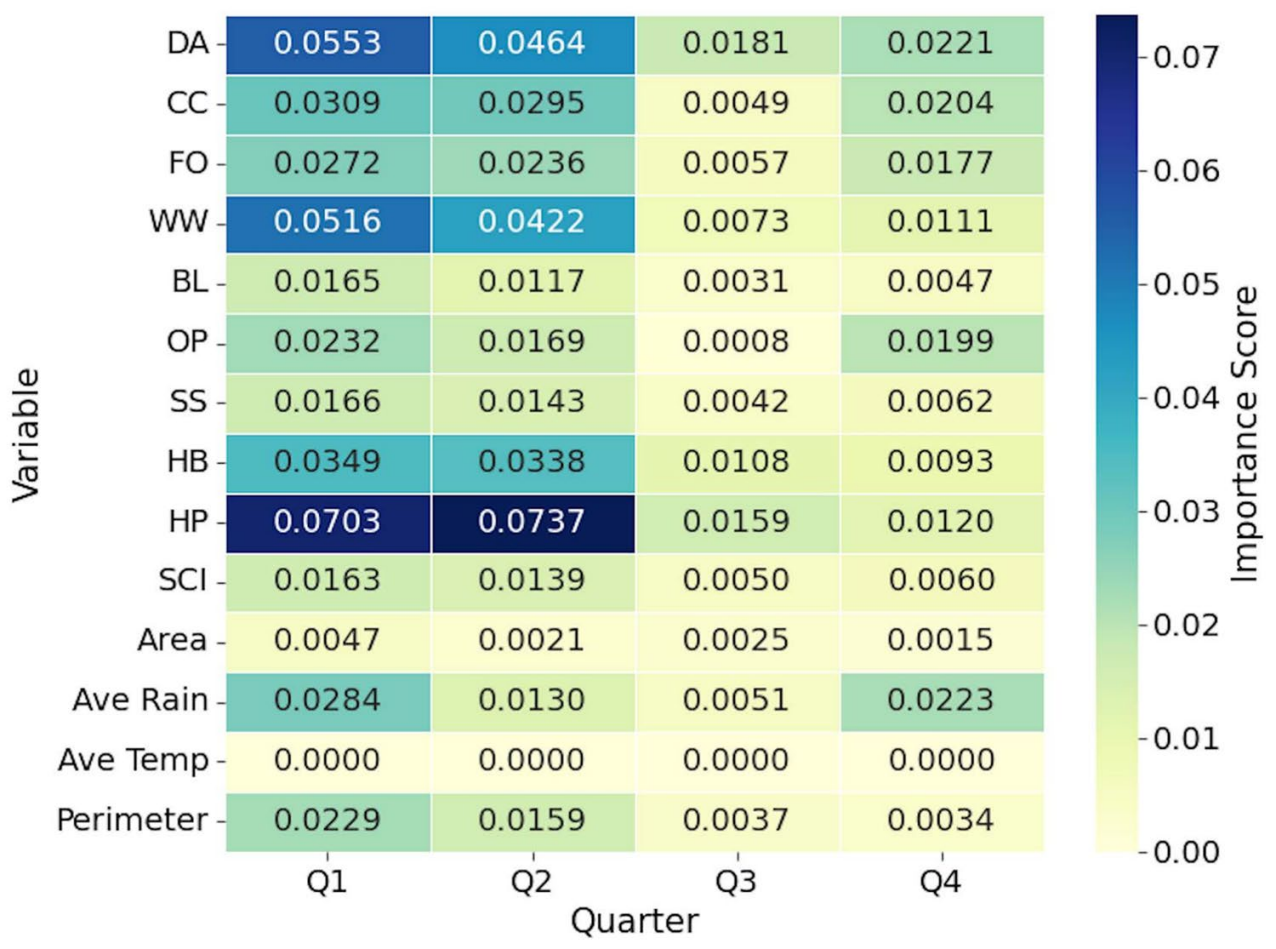
Heatmap showing the 4-year averaged importance scores by quarter in stage 1 for variables used in the estimation of P concentration. *DA* developed area, *CC* cultivated crop, *FO* forest, *WW* woody wetland, *BL* barren land, *OP* open water, *SS* shrub scrub, *HB* herbaceous, *HP* hay/pasture, *SCI* Shape Complexity Index.

**Fig. 6:**
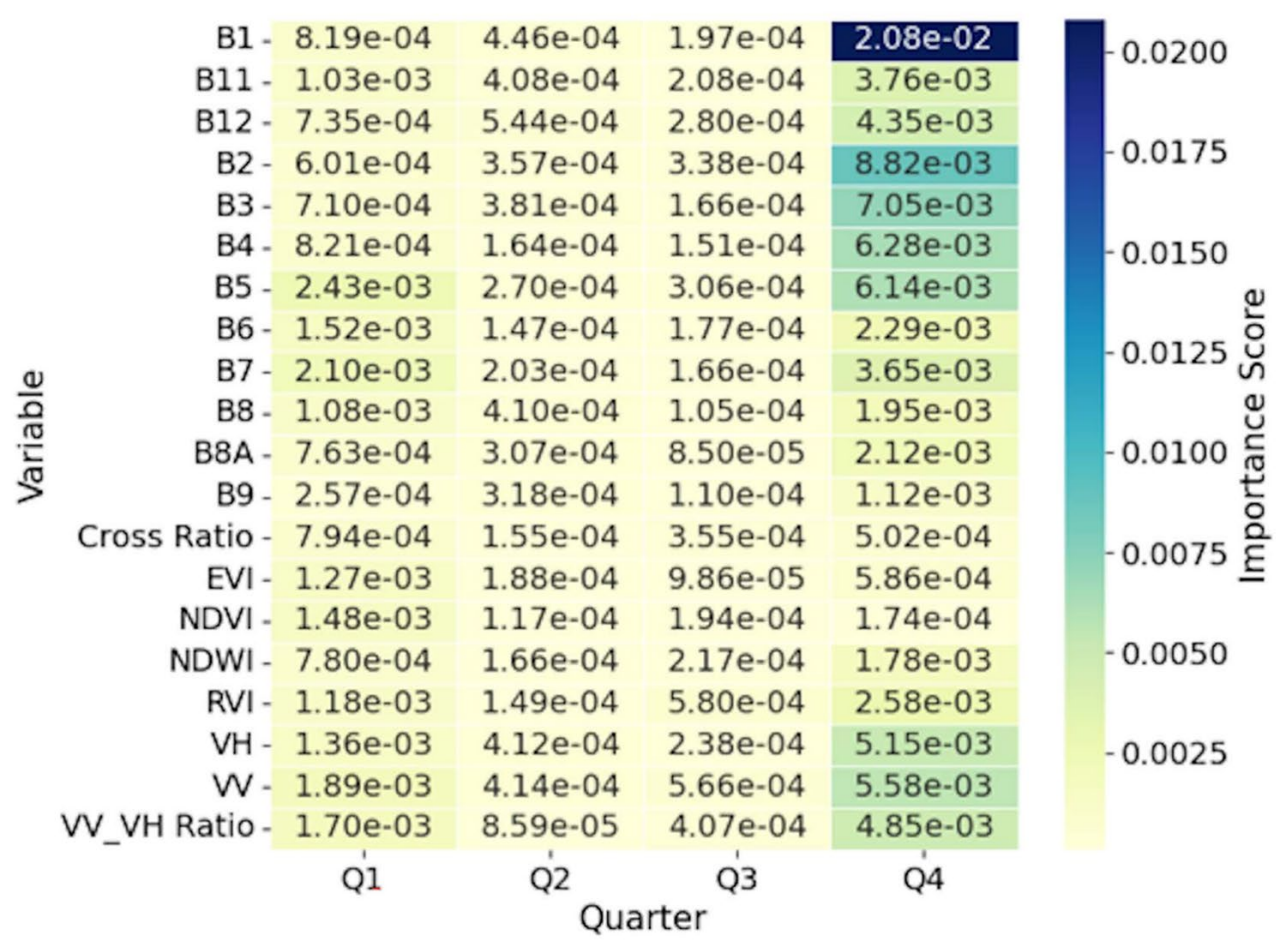
Heatmap showing the 4-year averaged importance scores by quarter in stage 2 for variables used in the estimation of P concentration. *B1 to B12* Sentinel-2 bands, *NDVI* Normalized Difference Vegetation Index, *NDWI* Normalized Difference Water Index, *EVI* Enhanced Vegetation Index, *RVI* Simplified Radar Vegetation Index, *VH* Vertical transmit/Horizontal receive, *VV* Vertical transmit/Vertical receive.

Analysis of the importance of RF variables revealed different predictive patterns between the concentrations of N (Figs. 3 and 4) and P (Figs. 5 and 6) in BWG, with landscape-level variables dominating both first-stage models but showing different magnitudes and seasonal dynamics. For N prediction, considering all quarters, herbaceous (HB) landuse class demonstrated the highest importance score, followed by woody wetland (WW), average rain, and hay/pasture (HP). At the same time, P models showed substantially lower peak importance values, with HP at the top of the list. The herbaceous class (HB) proved to be important for both nutrients. The developed class (DA) showed contrasting patterns, ranking lower for N (ranks 7, 4, 2, and 3 for Q1, Q2, Q3, and Q4, respectively) but achieving higher importance for P (rank 2, 2, 1, and 2 for Q1, Q2, Q3, and Q4, respectively). Agricultural variables exhibited nutrient-specific responses, with the cultivated crop (CC) showing moderate importance for the N but lower importance for the P model.

We observed pronounced seasonal variations for the hydrological and morphological variables, which differed between the two nutrients. The average rainfall showed strong temporal dynamics in the prediction of P, dropping from 0.028 in Q1 to 0.005 in Q3 before recovering to 0.022 in Q4, while maintaining a more consistent moderate importance for N (8.34). The class of open water (OP) exhibited contrasting seasonal patterns, showing minimal importance for P but substantial variation for N. The perimeter measurements showed stability across quarters for both nutrients, although with different magnitudes. The area measurements remained consistently low for both the N and P predictions. The Shape Complexity Index (SCI) maintained moderate importance for both nutrients, but showed greater stability in the P model compared to that of N.

The second stage analysis revealed different profiles of variable importance between nutrients. Spectral and water quality indices showed reduced predictive power for P compared to N. The importance of spectral bands varied substantially, with band 1 (B1) reaching its peak importance in Q4 for N and lower values for P. Other spectral bands (B2 to B12) generally exhibited importance scores for N but remained low for P across all quarters. The B8A band demonstrated consistent patterns for both nutrients; maintaining moderate and minimal importance for N and P, respectively.

## Discussion

We developed an RF framework that combined discrete site-specific variables with continuous RS data for wetland nutrient mapping. The success of our hybrid framework stemmed from its ability to (1) utilize the strengths of both discrete and continuous variable inputs, and (2) separate the predictive problem into two distinct and more manageable tasks without requiring extensive data transformation. Our final combined model showed higher predictions for N ($\text {r}^{2}$ = 0.90, RMSE = 0.129 mg/L) and P ($\text {r}^{2}$ = 0.89, RMSE = 0.007 mg/L) than the two individual stages. The improvement from stage 1 to the final combined model (N $\text {r}^{2}$ increased from 0.86 to 0.90; P $\text {r}^{2}$ increased from 0.86 to 0.89) validated our hypothesis that hybrid modeling could better capture the multiscale dynamics and processes of nutrients in wetlands. Guo et al.^20^ also observed an improved prediction accuracy when using a two-stage scheme to estimate heavy metal concentrations.

Xue et al.^22^ found comparable $\text {r}^{2}$ values of 0.85 to 0.87 for N and P prediction using RF regression with high-frequency sensor data. However, compared to our approach, we achieved higher accuracy by incorporating the spatial correction component. Similar to Giri and Qiu^23^ who emphasized the importance of landuse factors in predicting water quality, stage 1 results captured broad-scale patterns from the landscape characteristics and morphological features. Stage 2 then spatially corrected the baseline predictions and captured localized environmental variations not explained by stage 1 alone. Our approach addressed a critical limitation identified in previous wetland mapping and monitoring studies, where single-factor analyses often proved insufficient to capture complex nutrient dynamics^24,25^. Widely-used single-model approaches usually trains a single ML algorithm by combining a set of discrete and continuous predictors. Although powerful, this method could pose a challenge: the strong predictive signal from dominant, coarse-scale variables could overshadow the subtle, but critical, information contained within the high-resolution satellite imagery. Therefore, our two-stage hybrid framework was designed to overcome this limitation. By applying a two-stage approach, the interpretability of the model improved, such as a clearer understanding of the nutrient drivers.

Another advantage of our framework is its high performance when used on both nutrients on various time scales. The two-stage hybrid model accurately predicted nutrients with different biogeochemical behaviors ($\text {r}^{2}$ = 0.90 for N and $\text {r}^{2}$ = 0.89 for P). Over four years of study, the model maintained its accuracy despite seasonal variations. This result is important because it addresses the problem identified by Zhang et al.^2^, which is to develop a robust model appropriate for long-term monitoring. In addition, a model with consistent performance under different environmental conditions is also needed for effective management decisions^26^.

The variable importance analysis validated the conceptual basis of our two-stage approach. For N, the results showed a dual dependency on both stages of the model. The stage 1 model identified a competitive interaction between landuse considered nutrient sources (e.g. HP and CC)^27,28^ and landuse that served as buffers (e.g. WW and FO)^19,29,30^. This interaction highlighted the baseline N concentration as a function of landscape composition. However, the high importance of the stage 2 variables, namely vegetation indices (EVI, RVI) and SAR metrics (VV/VH ratio), showed that the final N concentration was affected by localized biophysical conditions in the wetland^31^. This two-part dependency showed consistency with the high solubility and biological reactivity of N.

In contrast to N, the prediction of P was dominated by the stage 1 model. This is in agreement with the results from Ding et al.^13^ where the distribution of P concentrations corresponded to the shore landuse. Although the most important predictor was the HP, the DA land use class was the most consistent in all quarters. We also saw this consistency of the DA for N. This result was expected since the DA had the highest percentage of landuse class contribution surrounding the sampling points (Supplementary Appendix 1). We found our results to be in agreement with studies linking P levels directly with anthropogenic landscapes^32,33^. The negligible predictive power of the stage 2 variables was an important finding. It suggested that once the land-use source signal has been explained in stage 1, very little remaining variance could be explained by the optical or radar properties of the water. This observation was aligned with the chemical nature of P that is less mobile and more particle-bound than N^34^. The weak signal detected in stage 2 was likely due to the turbidity of the water or suspended solids to which the P was adsorbed^35^.

The final nutrients maps provided a visual record of the ecological health of the wetlands. The N maps (Fig. 4) showed seasonal stress in the wetlands from diffuse agricultural sources (about 19% of the land use surrounding the wetlands is CC. The health of the wetlands depends on the buffering capacity provided by the contributing landscape (about 11% of the land use surrounding the wetlands is FO). The P maps (Fig. 5) showed a sustained decrease in concentrations from 2022 to 2024, particularly during the winter months of 2023 and 2024. We believe that the reduction in P levels was caused by the source loading rather than a change in in-stream processing. The variable importance analysis supported this interpretation.

The distinct maps that we generated for N and P confirmed the utility of our framework beyond a mere prediction. The framework diagnosed two distinct issues that impacted the health of the BWG wetlands. First, a diffuse N problem that is tied to landscape-scale processes and, second, a source-driven P problem where a significant reduction in loading has yielded a clear ecological improvement.

## Methods

**Fig. 7:**
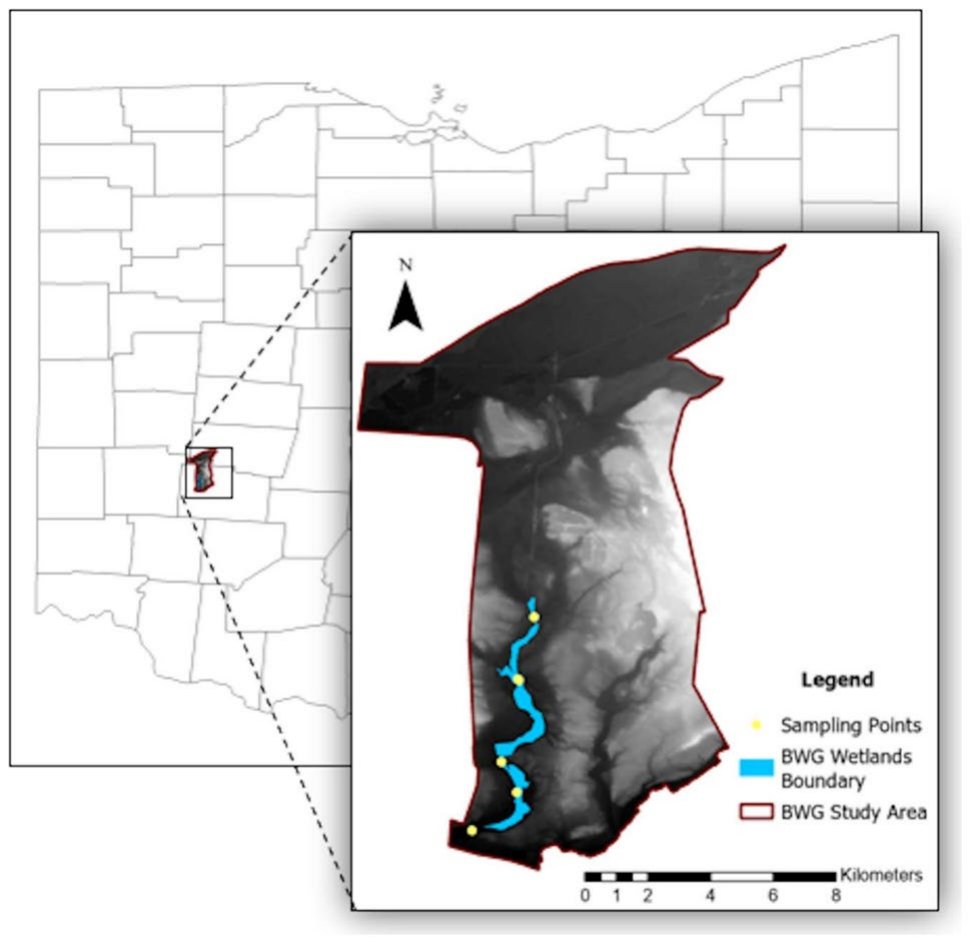
The BWG study area is in Beavercreek, Ohio, USA, showing the small-sized wetlands. This figure was created using ArcGIS Pro v.3.4.0 software by Esri (source: https://www.esri.com/).

This study focuses on three small wetlands (total area is about 2.0 km^2^) managed by Beavercreek Wetlands Greenway (BWG) Community Land Trust. Located in Beavercreek, Ohio, USA, the wetlands are protected by the Ohio Environmental Protection Agency (OEPA) Clean Water Act. Due to the variety of flora and fauna species, school groups, birdwatchers, and wildflower enthusiasts frequent the wetlands. The Beaver Creek stream runs through the wetlands from north to south. The BWG boundary and the perimeter of our study area are depicted in Fig. 7. According to NLCD land use, most urban areas (26.2%) of the BWG are in the east, while cultivated crops (38.2%) and forest areas (14.7%) predominate in the west.

**Fig. 8:**
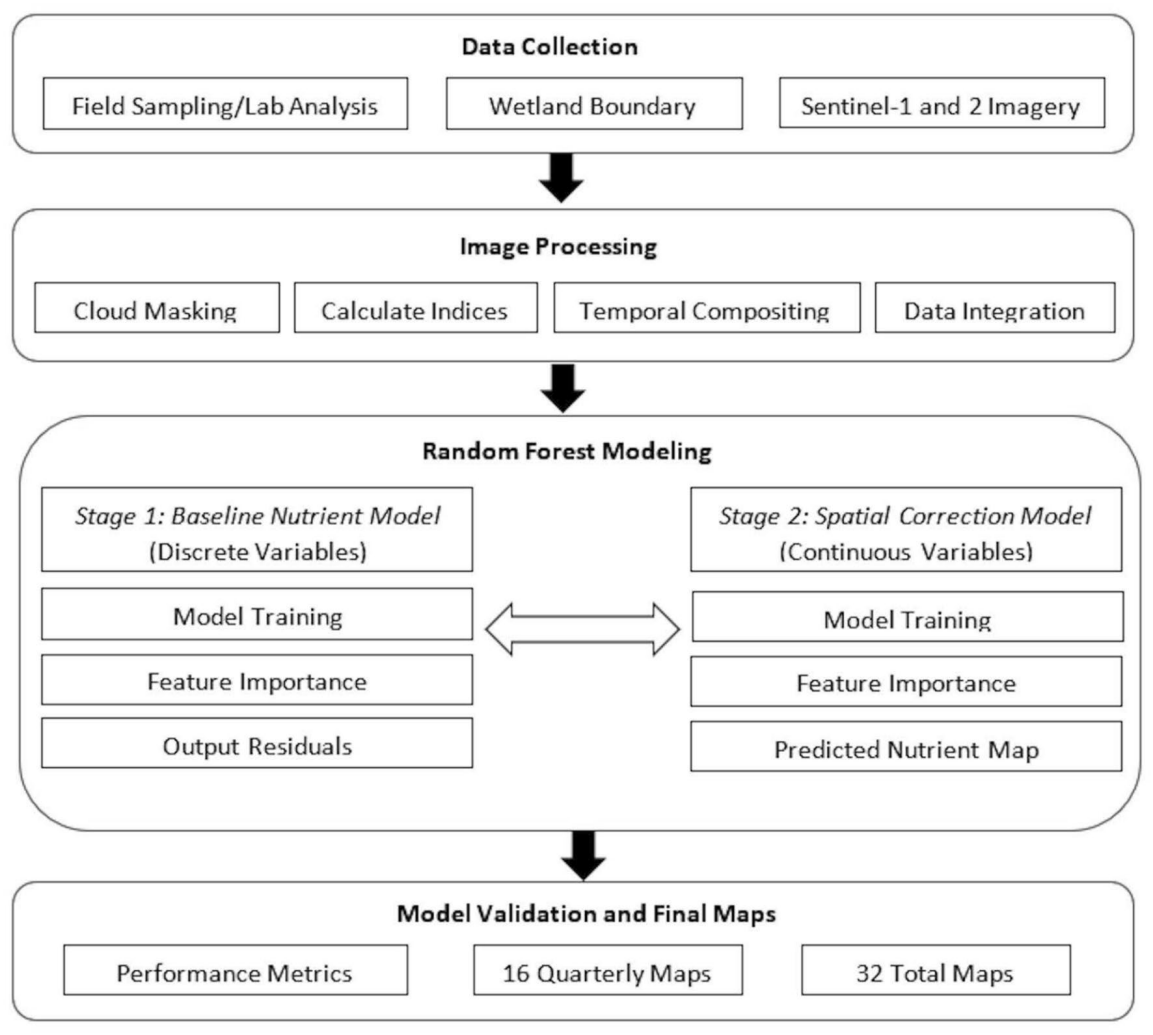
The four main components of the methodology include data collection, image processing, two-stage modeling using random forest, and evaluation/output. These components were performed within the Google Earth Engine (GEE) platform.

Figure 8 shows the two-stage hybrid modeling approach we used in the prediction and mapping of the BWG nutrients, N and P. Stage 1 models the baseline nutrient prediction using non-spatial wetland attributes. Next, stage 2 models the spatial error (residual) from the baseline model in stage 1 using satellite-derived environmental variables. This 2-step modeling strategy provides fine-scale spatial refinement to the final nutrient prediction and mapping. The main components of each stage include data collection, image processing, random forest modeling, and analysis/output. All major steps are performed within the Google Earth Engine (GEE) platform.

### Field sampling and laboratory analysis

We selected wetland sampling locations based on initial reconnaissance surveys. We considered factors such as the manageable size of the three wetlands ($<1.0 \, \text {km}^{2}$ each), their spatial distribution throughout the study area, ecological importance, and ease of accessibility for repeated field sampling. We then collected monthly water samples from representative locations within each wetland – inlet, middle, and outlet – to capture spatial variability in water quality parameters. We continuously collected monthly samples from 2021 to 2024 to capture seasonal nutrient fluctuations. During the 4-year study period, we obtained a total of 324 field water samples. In the laboratory, we filtered the samples and tested them for concentrations of nitrate (${\hbox {NO}}_{3}^{-}$) and orthophosphate (${\textrm{PO}}_4^{3-}$). Finally, we used the nutrient concentration values as input to the machine learning models to predict and spatially map the N and P distributions. The average concentrations of N and P are in Supplementary Appendices 2 and 3.

### Discrete variables and dataset

For the first stage of the modeling approach, we used several discrete characteristics of the wetlands as variable inputs to represent the physical, hydrological, and ecological properties of the wetland ecosystem (Table 1). We added these physical variables, namely, area and perimeter, to capture the basic morphometric characteristics that influence the cycle and retention capacity of nutrients^36^. We also calculated the Shape Complexity Index (SCI) and Perimeter-to-Area Ratio (PPS) to quantify the geometry of the wetland, as the configuration of the wetlands could impact on their ability to process nutrients^37,38^. We included climatic variables such as average temperature (AveTemp) and average rainfall (AveRain) since hydrological dynamics affects nutrient concentrations and these variables are considered high priorities in nutrient fluctuations in wetlands^39^.

In addition to morphological variables, we included landuse classes such as deciduous forest (DF), evergreen forest (EF), mixed forest (MF), shrub/scrub (SS), woody wetlands (WW), emergent herbaceous wetlands (EHW), herbaceous (HB), and hay/pasture (HP) to represent vegetation variables, different plant functional types, and specific nutrient uptake capacities^40^. To capture anthropogenic influences, we added cultivated crops (CC) and developed areas (DA) as both have a strong positive correlation with N and P nutrients and are considered sources of water pollutants^41^.

### Sentinel-1 and 2 satellite images, continuous variables and dataset

We used Sentinel-1 C-band Synthetic Aperture Radar (SAR) Ground Range Detected (GRD) data for our study period, available from the Sentinel Scientific Data Hub of the European Space Agency (ESA) (https://scihub.copernicus.eu/) to detect surface water quality^42^. We filtered the data to include only images captured in the Interferometric Wide Swath (IW) mode containing dual-polarization backscatter coefficients for VV (vertical transmit, vertical receive) and VH (vertical transmit, horizontal receive). To ensure radiometric consistency and stable viewing geometry, we selected a single orbital direction (either ascending or descending) based on the pass that offered more images. We converted the backscatter coefficients to a linear power scale for physical calculations.

From the selected SAR data, we derived the ratio of VV to VH, the simplified Radar Vegetation Index (SRVI), and the cross-polarization ratio (VH/VV) for each month of the study period (Table 2). SRVI is a robust measure of vegetation density that leverages the depolarization properties of volume scattering within a plant canopy^43^. We also produced a temporally averaged composite image by calculating the mean of all processed images. This new average image serves to reduce speckle noise and provides a representative measure of surface conditions during the study period. We utilized the final SAR-derived bands and indices^44^ as spatial predictors in stage 2 of our model (Fig. 8).

**Table 2:** List of the discrete and continuous variables used in the RF model, which consists of spectral indices, morphological properties, environmental factors, landuse classes, and the individual Sentinel-2 bands. The aVH represents the backscatter coefficient from the cross-polarization channel (vertical transmit, horizontal receive) in a linear power scale. The aVV represents the backscatter coefficient from the co-polarization channel (vertical transmit, vertical receive) in a linear power scale.

**Variable**	**Description**	**Equation/Notes**
**Continuous Variables**		
NDVI	Normalized Difference Vegetation Index	$\frac{NIR - Red}{NIR + Red}$
NDWI	Normalized Difference Water Index	$\frac{Green - NIR}{Green + NIR}$
EVI	Enhanced Vegetation Index	$2.5 \times \frac{NIR - Red}{NIR + 6 \times Red - 7.5 \times Blue + 1}$
B2	Sentinel-2 Blue Band (490 nm)	B2
B3	Sentinel-2 Green band (560 nm)	B3
B4	Sentinel-2 Red band (665 nm)	B4
B5	Sentinel-2 Red Edge 1 band (705 nm)	B5
B6	Sentinel-2 Red Edge 2 band (740 nm)	B6
B7	Sentinel-2 Red Edge 3 band (783 nm)	B7
B8	Sentinel-2 Near-Infrared (NIR) band (842 nm)	B8
B8A	Sentinel-2 Narrow NIR band (865 nm)	B8A
B11	Sentinel-2 Shortwave Infrared 1 (SWIR 1) band (1610 nm)	B11
B12	Sentinel-2 Shortwave Infrared 2 (SWIR 2) band (2190 nm)	B12
VV	Sentinel-1 SAR Co-polarization (Vertical transmit, Vertical receive)	VV
VH	Sentinel-1 SAR Cross-polarization (Vertical transmit, Horizontal receive)	VH
VV/VH Ratio	Ratio of VV to VH backscatter	$\frac{VV}{VH}$
SRVI	Simplified Radar Vegetation Index	$\frac{4(aVV)}{aVV + aVH}$
Cross Ratio	Cross-polarization ratio (VH / VV)	$\frac{VH}{VV}$
**Discrete Variables**		
SCI	Shape Complexity Index	$\frac{Perimeter}{Area}$
Area	Area for each wetland	–
Perimeter	Perimeter for each wetland	–
PPS	Polsby-Popper Score	$\frac{4\pi \times Area}{Perimeter^2}$
Ave Rain	Average 7-day precipitation for each wetland in mm	–
Ave Temp	Average 7-day temperature for each wetland in $^{\circ }$F	–
OP	Open Water	%OP
DA	Developed Open Space	%DOS
DA	Developed Low Intensity	%DLI
DA	Developed Medium Intensity	%DMI
DA	Developed High Intensity	%DHI
BL	Barren Land	%BL
FO	Deciduous Forest	%DF
FO	Evergreen Forest	%EF
FO	Mixed Forest	%MF
SS	Shrub/Scrub	%SS
HB	Herbaceous	%HB
HP	Hay/Pasture	%HP
CC	Cultivated Crops	%CC
WW	Woody Wetlands	%WW
EHW	Emergent Herbaceous Wetlands	%EHW

For Sentinel-2, we used the Level-2A (L2A) surface reflectance collection. Sentinel-2 has 13 spectral bands: four bands at 10 m resolution, six bands at 20 m resolution and three bands at 60 m spatial resolution. We resampled all bands that were 20 m resolution to 10 m to maintain consistency with the four native bands (B2 in blue, B3 in green, B4 in red, and B8 in NIR). We then computed the Normalized Difference Vegetation Index (NDVI), Enhanced Vegetation Index (EVI), and the Normalized Difference Water Index (NDWI). We based the selection of the spectral indices from previous studies that have applied ML to remote sensing data to predict nutrients for surface waters^45,46^. We also produced a cloud-reduced composite image by computing the per-pixel average of the entire time series. This composite, including the original spectral bands – band 2 (B2, 490 nm), band 3 (B3, 560 nm), band 4 (B4, 665 nm), band 5 (B5, 705 nm), band 6 (B6, 740 nm), band 7 (B7, 783 nm), band 8 (B8, 842 nm), band 8A (B8A, 865 nm), band 9 (B9, 945 nm), band 11 (B11, 1610 nm), and band 12 (B12, 2190 nm) – and the derived indices, represented the continuous variables from Sentinel-2.

When images (Sentinel-1 and Sentinel-2) were combined, GEE resampled the bands to a common projection and scale. This resampling process forced the Sentinel-1 data to align with the data grid of Sentinel-2 using the 10 m resolution. All of this image integration occurred at the feature level before the modeling stage. A complete list of continuous variables is found in Table 2. We used these variables as spatial predictors in the stage 2 model, in addition to the SAR-derived variables (Fig. 8).

### Random forest modeling

We utilized the Random Forest (RF) as the ML algorithm to predict and map nutrient concentrations^22,46,47^. RF is a popular model for predicting wetlands and other surface waters, including water quality parameters, using remote sensing data^38,48^. RF provides a way to select important covariates based on changes in prediction accuracy when variables are added or deleted from models. It is a non-parametric supervised classifier that uses the Classification and Regression Tree (CART) through bagging, where it randomly picks a set of features and creates a classifier with a bootstrapped sample of the training data to grow a tree^49^. With RF training data selection, it is possible that the same sample could be picked several times, whereas others may not be picked at all. Apart from RF being quite robust with highly collinear variables, the random selection process at each tree node causes low correlation among trees and avoids overfitting^50^.

We guaranteed a precise alignment of the variables before applying the RF model. For temporal alignment, first, we grouped the in-situ nutrient samples for that particular quarter (e.g., Q1-2021: January 1 through March 31). Next, we used all available Sentinel-1 and Sentinel-2 images that were collected in the same quarter to generate one mean composite image. This part of our methodology was crucial because (1) it provides a strong representation of the average environmental conditions during the period when the samples were taken, and (2) it minimizes the impact of atmospheric conditions or day-to-day variability, thus, our process makes a more stable set of predictors. We ensured that every point sample was temporally matched to the mean environmental state of its corresponding quarter. In terms of spatial alignment, our model extracted the corresponding pixel values from the quarterly mean composite image for each in-situ sample point through its geographic coordinates. This part of our model ensured that each sample point was precisely linked to a single vector of predictor values that represents the pixel area where the sample was collected.

#### Two-stage hybrid RF modeling framework

**Fig. 9:**
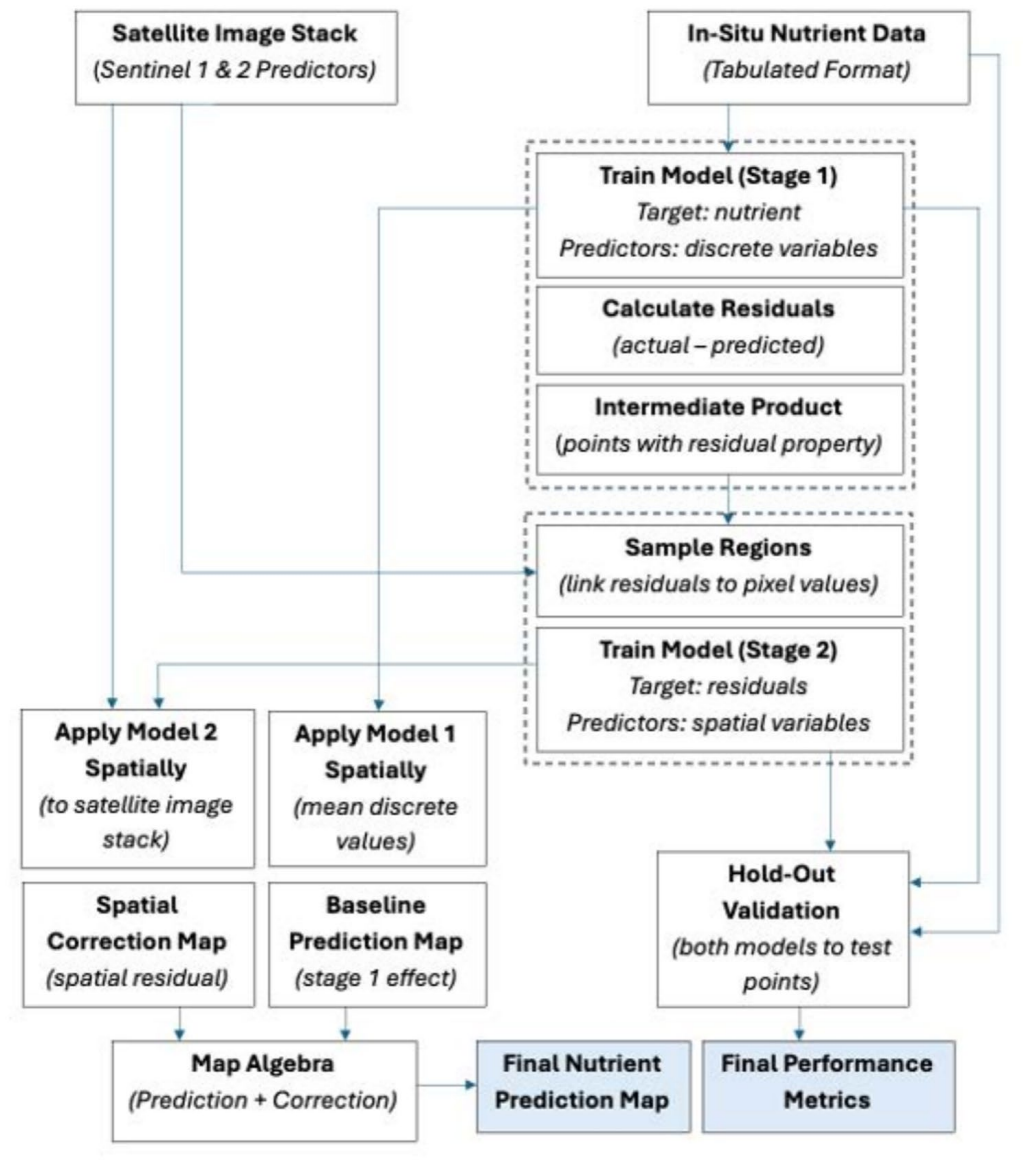
The detailed two-stage RF hybrid modeling framework, illustrating the integration of discrete data (stage 1) and satellite imagery (stage 2) to generate a final nutrient prediction map.

In this study, we employed a two-stage hybrid RF regression framework designed to combine discrete, site-specific variables (stage 1) with continuous and spatially explicit RS data (stage 2). The first stage used an RF model to establish a baseline nutrient prediction as a function of the discrete wetland characteristics (e.g., area, shape, climate). The model was trained using 70% of the in-situ data points. The output of the stage 1 model are the residuals or the difference between observed and predicted values. These values captured the variance not explained by the site-level variables. Next, we used these residuals as explicit target variables for the next stage. In stage 2, we employed another independent RF model to explain the residual variance using the continuous predictors derived from Sentinel-1 SAR and Sentinel-2 imagery. For mapping the spatial distribution of the nutrients, the stage 1 model generated a general baseline prediction map. In contrast, the stage 2 model produced a detailed correction map on a per-pixel basis. Finally, we generated a nutrient prediction map by summing the baseline prediction and the spatial correction maps using map algebra. This last step of adding maps created a product that uses both the general trends from physical site data and the localized environmental variations captured by RS. Figure 9 shows a more detailed diagram of the two-stage modeling framework.

#### Model validation

To quantitatively assess the predictive performance of our model, we performed a validation on an independent hold-out dataset. We formed the test set by randomly partitioning 30% of the original data points, and the rest was used to train or adjust the model. This approach guaranteed that the performance of our model is tested on a set of data that was not used during the training phase. We evaluated the performance of the combined two-stage model using a list of statistical metrics^51^: (1) Coefficient of Determination ($\text {r}^{2}$) to quantify the proportion of the variance in the observed nutrient concentrations that is explained by the model; (2) Root Mean Square Error (RMSE) and the Mean Absolute Error (MAE) to measure the magnitude of prediction error; and (3) Mean Bias Error (MBE) to diagnose any systematic tendency of the model to over-predict (positive bias) or under-predict (negative bias). This set of metrics ensures a general understanding of the performance of our model and how the model captured the goodness-of-fit, magnitude of error, and directional bias^52^.

### Google Earth engine

We ran the hybrid RF framework through the GEE platform (earthengine.google.com). GEE is a cloud-based platform that provides researchers with a vast collection of satellite imagery and geospatial datasets^53^. It offers a comprehensive and diverse range of data sources, including Sentinel-1 SAR and Sentinel-2 Level-2A (L2A) images. The extensive GEE data archive, coupled with its efficient data storage and processing capabilities, allowed this research seamless access to the necessary image data for analysis. Using the power of GEE, we retrieved and preprocessed the images for all the months and years required for this study. The use of GEE ensured a streamlined and efficient workflow, enabling us to take advantage of its vast data resources and advanced analysis capabilities for accurate nutrient mapping^54^.

## Implications

The findings of this study have implications for wetland management and nutrient cycling studies. We demonstrated that our model could predict N and P nutrients using a dynamic, robust, and spatially comprehensive two-stage approach. The quarterly nutrient mapping provides valuable information for the rapid identification of hotspots and assessment of management interventions.

The ability of our model to distinguish between N and P drivers could help target management strategies in wetlands. For instance, to improve P concentrations, specific point sources have to be addressed, urban runoff policies have to be implemented, or buffer zones have to be established around DA to reduce direct runoff inputs. For N management, landscape-scale implementation of agricultural best management practices is required. One practice that can be implemented is cover cropping and nutrient management plans in surrounding farm fields.

From a scientific perspective, this study provides a framework for integrating diverse data sources to model complex environmental processes. The two-stage hybrid framework enhances ecological interpretability through the quantitative separation of localized biophysical processes and broad source patterns. Our modeling approach demonstrates high transferability and could be adapted to model additional water quality parameters, such as total suspended solids. Lastly, our framework does not only apply for current wetland monitoring. It also has the potential to assess the impacts of future development on wetland water quality.

## Caveats and future research directions

We recognize that our two-stage hybrid framework has inherent limitations. Below, we list these limitations so that we can set directions for further investigation.

1. The BWG is small and has unique hydrological and biogeochemical conditions, which were used to calibrate the model. To apply the model to other wetland systems with larger areas, different climates, geologies, or percentage of land-use contributions, local training data and recalibration are needed to ensure precision.
2. Our model is useful for capturing seasonal trends and is based on the quarterly temporal composites of the satellite image. However, this could mitigate the effects of high intensity short-term events, such as large storms, which could affect nutrient transport. To better capture these event-based dynamics, future research could utilize high-temporal monthly data.
3. Our model is only sensitive to surface conditions. RS-based models do not have the capability to measure processes that occur deeper in the water column. A future study is necessary to understand these subsurface processes through the integration of our current framework with process-based hydrological models.
4. The level or depth of the water is a factor in changes in nutrient concentrations in wetlands through dilution and concentration mechanisms. For future studies, we recommend adding in situ water level data as one of the input variables.
5. Lastly, although our model successfully highlighted the factors that influence nutrient change, the increase in nutrient levels in some seasons needs a focused examination to determine the root cause. In order to transform our predictive framework into a comprehensive and globally applicable tool for wetland monitoring, it will be important to address these limitations.

## Conclusion

This study successfully created and validated a two-stage hybrid modeling framework for high-resolution spatiotemporal mapping of nutrient concentrations in wetland ecosystems. The predictive accuracy of the model for both nutrients (N and P) highlighted its ability to integrate continuous satellite imagery with discrete site-level datasets. Being multi-seasonal, our framework allowed us to move beyond a single static model. Our results showed which seasons are the most suitable for remote sensing-based nutrient monitoring. Also, our analysis identified divergent biogeochemical pathways for each nutrient. Source loading from DA landuse class dominated P concentrations, while seasonal agricultural pressures and in-stream processing affected N dynamics. Finally, this study offers a strong diagnostic framework that could inform focused wetland management strategies and monitor the health and function of wetland ecosystems.

## Supplementary Information

Supplementary Information 1.

Supplementary Information 2.

Supplementary Information 3.

## Data Availability

The datasets generated during and/or analyzed during the current study are available from the corresponding author upon reasonable request.
