# Supplementary material for: Hybrid spatiotemporal modeling of nutrient cycling in wetland ecosystems using advanced mapping techniques and machine learning approaches
Source: Sci Rep. 2026 Feb 19;16:9954. doi: 10.1038/s41598-026-40585-5 (PMC13022218; doi:10.1038/s41598-026-40585-5)
Supplement: Supplementary file 1 — Supplementary Information 1. [file 41598_2026_40585_MOESM1_ESM.docx]

**Appendix 1:** Percent contribution of each landuse class for each main sampling point (inlet and outlet). We used hydrological analysis to compute each contributory area. OP = open water, DA = developed, BL = barren land, FO = forest, SS = shrub scrub, HB = herbaceuous, HP = hay/pasture, CC = cultivated crops, WW = woody wetlands, EHW = emergent herbaceuous wetlands.

| Point | OP | DA | BL | FO | SS | HB | HP | CC | WW | EHW |
| --- | --- | --- | --- | --- | --- | --- | --- | --- | --- | --- |
| 1 | 0.4 | 57.0 | 0.0 | 5.5 | 0.0 | 0.0 | 12.6 | 20.3 | 3.6 | 0.6 |
| 2 | 0.0 | 49.3 | 0.0 | 6.4 | 0.0 | 0.0 | 16.6 | 23.7 | 2.3 | 1.6 |
| 3 | 0.0 | 56.5 | 0.0 | 15.7 | 0.0 | 0.0 | 7.1 | 16.8 | 2.2 | 1.6 |
| 4 | 0.0 | 33.1 | 0.0 | 17.4 | 0.0 | 0.1 | 19.4 | 28.5 | 1.2 | 0.3 |
| 5 | 0.3 | 58.9 | 0.5 | 4.9 | 0.3 | 0.3 | 23.0 | 7.5 | 1.2 | 3.2 |
| 6 | 0.4 | 47.9 | 1.4 | 14.6 | 0.2 | 0.2 | 15.6 | 18.8 | 0.4 | 0.5 |
