# Supplementary material for: Hybrid spatiotemporal modeling of nutrient cycling in wetland ecosystems using advanced mapping techniques and machine learning approaches
Source: Sci Rep. 2026 Feb 19;16:9954. doi: 10.1038/s41598-026-40585-5 (PMC13022218; doi:10.1038/s41598-026-40585-5)
Supplement: Supplementary file 2 — Supplementary Information 2. [file 41598_2026_40585_MOESM2_ESM.docx]

**Appendix 2**: The quarterly average of nitrate concentration each year for the sampling points.

|  |  | 1 | 2 | 3 | 4 | 5 | 6 | 7 | 8 | 9 |
| --- | --- | --- | --- | --- | --- | --- | --- | --- | --- | --- |
| 2021 | Q1 | 0.8222 | 1.0510 | 1.2798 | 0.9892 | 0.7770 | 0.5648 | 0.5395 | 0.5942 | 0.6488 |
|  | Q2 | 0.6367 | 0.6167 | 0.6264 | 0.5150 | 0.4663 | 0.2687 | 0.2687 | 0.2477 | 0.5507 |
|  | Q3 | 0.4818 | 0.4361 | 0.4338 | 0.4788 | 0.4758 | 0.2784 | 0.2388 | 0.3041 | 0.3820 |
|  | Q4 | 0.6431 | 0.6518 | 0.6294 | 0.5131 | 0.0226 | 0.2102 | 0.2537 | 0.6958 | 0.0946 |
| 2022 | Q1 | 0.5683 | 0.5763 | 0.5449 | 0.5339 | 0.0894 | 0.5699 | 0.6669 | 0.7039 | 0.1493 |
|  | Q2 | 0.6574 | 0.6664 | 0.6774 | 0.5554 | 0.0092 | 0.2497 | 0.2537 | 0.5697 | 0.0321 |
|  | Q3 | 0.8023 | 0.8190 | 0.8550 | 0.6547 | 0.0133 | 0.0859 | 0.0775 | 0.3083 | 0.0136 |
|  | Q4 | 0.8028 | 0.8255 | 0.8105 | 0.7741 | 0.0078 | 0.3050 | 0.3212 | 1.1091 | 0.0357 |
| 2023 | Q1 | 0.5559 | 0.5909 | 0.5649 | 0.6316 | 0.0075 | 0.5079 | 0.5419 | 0.6253 | 0.1576 |
|  | Q2 | 0.6026 | 0.6116 | 0.6429 | 0.5439 | 0.0061 | 0.3441 | 0.3128 | 0.2659 | 0.0117 |
|  | Q3 | 0.7075 | 0.5955 | 0.7585 | 0.6772 | 0.0055 | 0.0935 | 0.0884 | 0.2965 | 0.0263 |
|  | Q4 | 0.7571 | 0.7611 | 0.7928 | 0.6751 | 0.0076 | 0.2908 | 0.3415 | 0.6458 | 0.0667 |
| 2024 | Q1 | 0.4775 | 0.4878 | 0.4832 | 0.3498 | 0.1226 | 0.4578 | 0.4662 | 0.5555 | 0.1347 |
|  | Q2 | 0.5873 | 0.4406 | 0.6253 | 0.5982 | 0.0064 | 0.2512 | 0.2289 | 0.3292 | 0.0050 |
|  | Q3 | 0.7262 | 0.4713 | 0.8424 | 0.6725 | 0.0022 | 0.0426 | 0.0392 | 0.0024 | 0.0016 |
|  | Q4 | 0.6654 | 0.6630 | 0.7003 | 0.5619 | 0.0000 | 0.2964 | 0.3036 | 0.0486 | 0.3507 |
