# Supplementary material for: Hybrid spatiotemporal modeling of nutrient cycling in wetland ecosystems using advanced mapping techniques and machine learning approaches
Source: Sci Rep. 2026 Feb 19;16:9954. doi: 10.1038/s41598-026-40585-5 (PMC13022218; doi:10.1038/s41598-026-40585-5)
Supplement: Supplementary file 3 — Supplementary Information 3. [file 41598_2026_40585_MOESM3_ESM.docx]

**Appendix 3**: The quarterly average of orthophosphate concentration each year for the sampling points.

|  |  | 1 | 2 | 3 | 4 | 5 | 6 | 7 | 8 | 9 |
| --- | --- | --- | --- | --- | --- | --- | --- | --- | --- | --- |
| 2021 | Q1 | 0.0077 | 0.0264 | 0.0452 | 0.0043 | 0.0047 | 0.0051 | 0.0041 | 0.0046 | 0.0052 |
|  | Q2 | 0.0649 | 0.0486 | 0.0327 | 0.0504 | 0.0389 | 0.0314 | 0.0388 | 0.0367 | 0.0368 |
|  | Q3 | 0.0143 | 0.0083 | 0.0051 | 0.0082 | 0.0228 | 0.0050 | 0.0080 | 0.0031 | 0.0123 |
|  | Q4 | 0.0299 | 0.0311 | 0.0246 | 0.0120 | 0.0120 | 0.0074 | 0.0099 | 0.0048 | 0.0103 |
| 2022 | Q1 | 0.0198 | 0.0166 | 0.0598 | 0.0151 | 0.0151 | 0.0143 | 0.0162 | 0.0116 | 0.0089 |
|  | Q2 | 0.0387 | 0.0129 | 0.0163 | 0.0135 | 0.0157 | 0.0132 | 0.0132 | 0.0109 | 0.0067 |
|  | Q3 | 0.0284 | 0.0307 | 0.0293 | 0.0278 | 0.0390 | 0.0162 | 0.0212 | 0.0158 | 0.0289 |
|  | Q4 | 0.0130 | 0.0121 | 0.0155 | 0.0137 | 0.1092 | 0.0111 | 0.0105 | 0.0097 | 0.0131 |
| 2023 | Q1 | 0.0149 | 0.0142 | 0.0147 | 0.0130 | 0.0163 | 0.0106 | 0.0083 | 0.0076 | 0.0077 |
|  | Q2 | 0.0130 | 0.0134 | 0.0104 | 0.0116 | 0.0109 | 0.0085 | 0.0075 | 0.0057 | 0.0070 |
|  | Q3 | 0.0136 | 0.0162 | 0.0143 | 0.0190 | 0.0214 | 0.0109 | 0.0088 | 0.0052 | 0.0109 |
|  | Q4 | 0.0159 | 0.0147 | 0.0160 | 0.0169 | 0.0205 | 0.0232 | 0.0147 | 0.0080 | 0.0228 |
| 2024 | Q1 | 0.0161 | 0.0204 | 0.0173 | 0.0134 | 0.0097 | 0.0076 | 0.0058 | 0.0063 | 0.0061 |
|  | Q2 | 0.0040 | 0.0027 | 0.0074 | 0.0096 | 0.0144 | 0.0071 | 0.0042 | 0.0036 | 0.0018 |
|  | Q3 | 0.0000 | 0.0006 | 0.0010 | 0.0029 | 0.0087 | 0.0073 | 0.0061 | 0.0000 | 0.0052 |
|  | Q4 | 0.0051 | 0.0050 | 0.0076 | 0.0066 | 0.0218 | 0.0022 | 0.0108 | 0.0137 | 0.0070 |
